# Action of Chloral on the Insane

**Published:** 1870-10

**Authors:** 


Action of Chloral on the Insane.—Dr. J. B. Tuke, Supt., &c., Lunatic Asylum, has instituted some experiments to determine the effect of hydrate of chloral in the treatment of the insane. He selected, upon which to experiment, a case of chronic alcoholism, with acute mania; two cases of asthemic insanity, with melancholy; and a case of climacteric insanity, accompanied with melancholy.

These patients were carefully observed for three days previous to the use of the chloral, and the temperature, pulse, respiration, and general condition were carefully noted. For the next four days, half-drachm doses of the medicine were given, morning and night, the effect being noted. On the next three days, the medicine was omitted, when it was again administered for four days. Subsequently half-drachm doses were given at night, for a few nights, and then entirely discontinued.

In the first case much improvement followed the use of the remedy; the patient was much quieter, and sleep was improved. She had been very maniacal for weeks before this treatment, but after it, became conscious, went to work, and slept in the general dormitory.

With the second patient, after the treatment there was "well-marked improvement in the case."

Of the third patient, he says, after the chloral had been given for a time, "her mental symptoms were much abated. She began to work, and, although still miserable, to see some hope for herself. On discontinuing the chloral, restlessness and irritability returned, which were again allayed on its resumption." The medicine was then given four days, with good results, after which it was declared that she was well on the road to convalescence, that she was employed as a housemaid, slept well, and was rapidly gaining in bodily condition.

The fourth case was a remarkable one. The patient was noisy, restless, and dementedly melancholy; her case was regarded as almost hopeless.

The effect of the chloral on this case was striking; a sleep followed each dose. On leaving off the medicine temporarily, the old symptoms—which, while the drug was being taken, were held in abeyance—returned, but with less severity. When resumed, it produced the same effect as at first; on its second discontinuance the condition of the patient was much improved.

Dr. T. has used it frequently in chronic cases of insanity, in which violent outbursts of excitement occur, and always with good effect.

The insomnia of climacteric melancholy is much relieved by a dose of 30 or 40 grains at bedtime.

He regards it as the most valuable means of procuring sleep now in use by asylum physicians.

There is some difficulty in ascertaining the exact dose for each case; but this is obviated by beginning with half-drachm doses, and increasing till the limit is found.—*Lancet.*

